# Polyacrylamide-Based Antimicrobial Copolymers to Replace or Rescue Antibiotics

**DOI:** 10.1021/acscentsci.4c01973

**Published:** 2025-03-13

**Authors:** Shoshana C. Williams, Madeline B. Chosy, Carolyn K. Jons, Changxin Dong, Alexander N. Prossnitz, Xinyu Liu, Hector Lopez Hernandez, Lynette Cegelski, Eric A. Appel

**Affiliations:** † Department of Chemistry, Stanford University, Stanford, California 94305, United States; ‡ Sarafan ChEM-H, Stanford University, Stanford, California 94305, United States; § Department of Materials Science & Engineering, Stanford University, Stanford, California 94305, United States; ∥ Department of Bioengineering, Stanford University, Stanford, California 94305, United States; ⊥ Woods Institute for the Environment, Stanford University, Stanford, California 94305, United States; # Department of Pediatrics (Endocrinology), Stanford University, Stanford, California 94305, United States

## Abstract

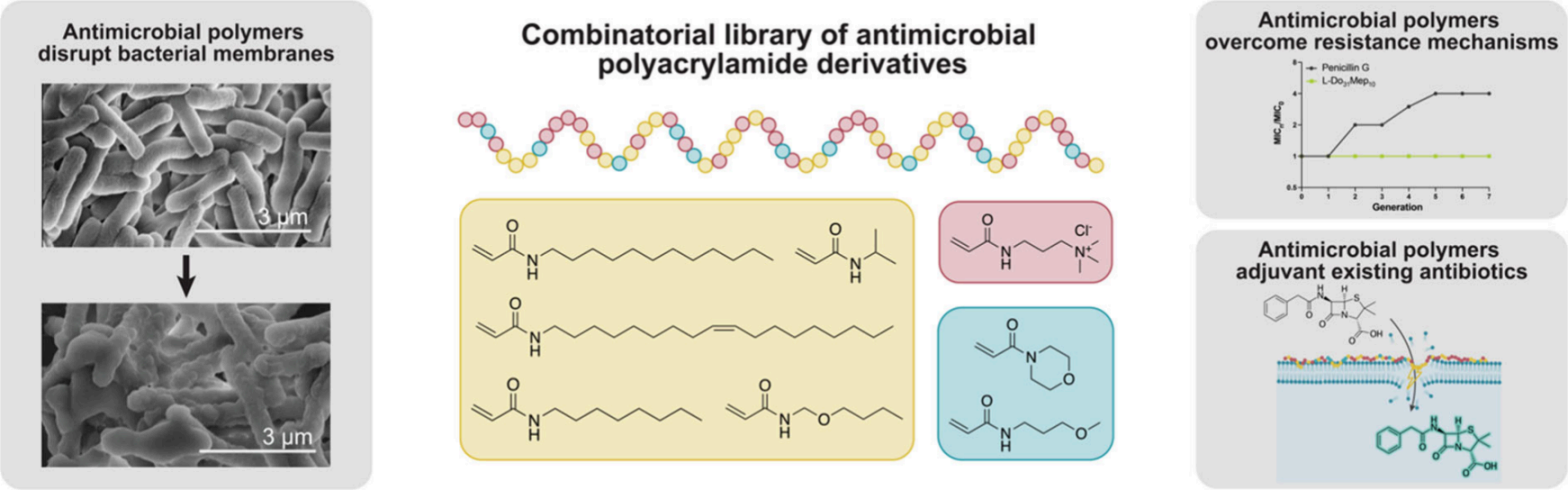

Antibiotics save countless lives each year and have dramatically improved human health outcomes since their introduction in the 20th century. Unfortunately, bacteria are now developing resistance to antibiotics at an alarming rate, with many new strains of “superbugs” showing simultaneous resistance to multiple classes of antibiotics. To mitigate the global burden of antimicrobial resistance, we must develop new antibiotics that are broadly effective, safe, and highly stable to enable global access. In this manuscript, we report the development of polyacrylamide-based copolymers as a class of broad-spectrum antibiotics with efficacy against several critical pathogens. We demonstrate that these copolymer drugs are selective for bacteria over mammalian cells, indicating a favorable safety profile. We show that they kill bacteria through a membrane disruption mechanism, which allows them to overcome traditional mechanisms of antimicrobial resistance. Finally, we demonstrate their ability to rehabilitate an existing small-molecule antibiotic that is highly subject to resistance development by improving its potency and eliminating the development of resistance in a combination treatment. This work represents a significant step toward combating antimicrobial resistance.

## Abstract 2

Polyacrylamide-based copolymers function as broad-spectrum antibiotics via a membrane disruption mechanism. They can prevent or delay the onset of resistance and rehabilitate existing antibiotics.

## Introduction

**Figure 1:**
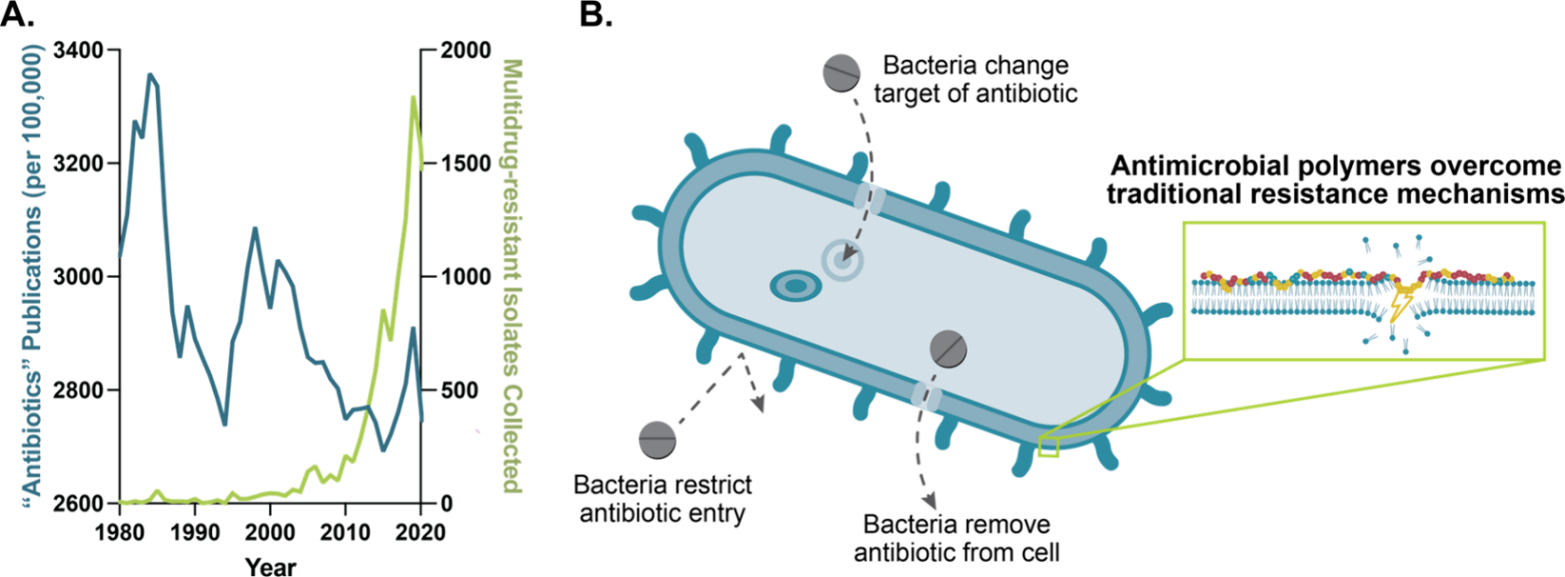
Polymers to combat antimicrobial resistance. (A) Graph comparing the decreasing research into antibiotics with the increasing incidence of MDR bacteria. Values determined using NCBI tools: PubMed by Year and MicroBIGG-E. (B) Schematic showing common mechanisms of AMR and the hypothesized ability of copolymers to overcome these mechanisms.

Antimicrobial resistance (AMR) is a growing global crisis. It is estimated that by 2050, over 8.2 million deaths annually will be associated with AMR.^1^ In the US alone, AMR already causes over 35,000 deaths annually and costs over $4.6 billion in direct healthcare spending.^2,3^ Unfortunately, progress in the development of new antibiotics has waned, while AMR bacteria arise at an alarming rate.^4,5^ Even as the identification of multidrug resistant (MDR) isolates increases,^6^ publications on antibiotics are decreasing (Figure 1A).^7^

The mechanisms of AMR are molecularly defined and include inhibition of drug uptake, modification of the drug target, inactivation of the drug, and enhanced drug efflux (Figure 1B).^8,9^ Novel antibiotics that could evade these mechanisms are of immense interest to combat the growing threat of AMR.

Previous work has explored the development of antimicrobial polymers and oligomers, both as coatings for devices or surfaces^10−18^ and as treatments.^19−49^ These studies have identified positive charge and hydrophobicity as key parameters to enable antibacterial activity, and several of these polymers were shown to work through a membrane disruption mechanism.^50−64^ This mode of action overcomes traditional resistance mechanisms, since the polymers do not need access to the intracellular space, and they are not specific to a singular molecular target where mutations confer resistance (Figure 1B).^65^ Despite these exciting developments, the further translation of these polymers has been hampered by challenging synthesis and toxicity by hemolytic activity. Several approaches have relied on multistep syntheses to prepare monomers and perform postpolymerization modifications, which can be costly and time-intensive.^29,30,35−38,45,46,63^ Other investigations have utilized sequence-controlled polymers, which similarly require greater synthetic control.^28,31−34,66^ Even with these approaches, several candidates have shown unfavorable hemolytic activity and toxicity profiles.^29,33−36,67^ To address these challenges, we generated a library of polyacrylamide-derived copolymers for use as antimicrobial agents. Polyacrylamides were selected for their impressive chemical stability, commercial availability, and ease of synthesis. We investigated their activity, safety, mechanism of action, and ability to rescue the effectiveness of existing small-molecule antibiotics.

## Results and Discussion

### Development of a Library of Polyacrylamide Derivative Copolymers

**Figure 2:**
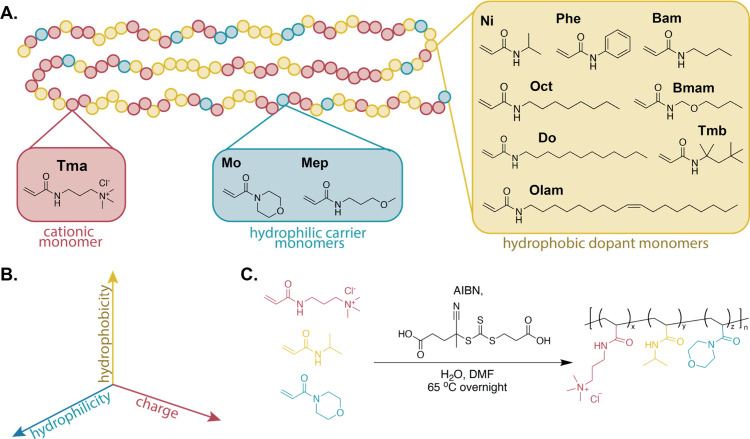
Development of a library of polyacrylamide-based copolymers. (A) Schematic of statistical copolymer library showing monomers used and their classes. (B) The use of a ternary system allows for independent tuning of charge and hydrophobicity. (C) RAFT polymerization conditions.

We developed a library of unique polyacrylamide derivatives designed to arrest bacterial growth through a membrane disruption mechanism, which does not rely on specific protein transporters or pathways subject to resistance mechanisms. Each copolymer comprises various ratios of three monomers, including: (i) a cationic monomer to drive polymer adsorption to the negatively charged surface of bacteria, (ii) a hydrophilic “carrier” monomer to tune water solubility, and (iii) a hydrophobic “dopant” monomer to disrupt the bacterial membrane (Figure 2A). Each polymer contained the same cationic monomer, (3-acrylamidopropyl)trimethylammonium chloride (Tma). We designed this library to vary hydrophobic and hydrophilic monomer identity, charge density, hydrophobicity, and molecular weight to allow us to explore how these parameters affect activity and safety. Inclusion of a third monomer allowed for greater control in exploring the independent effects of charge and hydrophobicity (Figure 2B). It also allowed for control of hydrophilicity, which is known to impact antimicrobial activity and biocompatibility.^22,68^ Previous work in the field has explored these parameters through the development of polymers where the hydrophobicity and charge were linked, either in the same monomers or using two monomers.^10,28,35,36^

Copolymers were synthesized through statistical copolymerization using a variety of commercially available or easily synthesized monomers by reversible addition–fragmentation chain transfer (RAFT) polymerization (Figure 2C). Each polymer is named by its target degree of polymerization (DP; H for a high DP of 115; L for a low DP of 70), and the identity and target weight percent of its hydrophobic and hydrophilic monomers. The remaining weight percent is accounted for by the cationic monomer, which is (3-acrylamidopropyl)trimethylammonium chloride) in all cases. For example, L-Ni_31_Mo_10_ has a low degree of polymerization (70) and consists of (3-acrylamidopropyl)trimethylammonium chloride (59%), *N*-isopropylacrylamide (31%), and 4-acryloylmorpholine (10%). The copolymers were analyzed by nuclear magnetic resonance (NMR; Figures S3–S10). For several polymers, their conversion and composition were recorded (Table 1, Table S1). The observed compositions closely matched the target molar ratios of monomers. A computational analysis, using the previously reported program Compositional Drift, was performed to analyze compositional variance for a subset of polymers (Figures S11, S12).^69−71^

**Table 1:** Composition of Polyacrylamide Library

Polymer	Target DP^a^	Target M_n_ (kDa)^a^	M_n_ (kDa)^b^	Đ^b^	% Conversion^c^	% Yield^d^
L-Ni_31_Mo_10_	70	11.1	9.9	1.12	n.d.	103
L-Ni_31_Mep_10_	70	11.1	10.6	1.12	n.d.	113
L-Phe_31_Mo_10_	70	12.4	8.9	1.16	n.d.	94.5
L-Phe_31_Mep_10_	70	12.4	9.2	1.14	n.d.	99
L-Do_31_Mo_10_	70	14.4	7.6	1.36	n.d.	92.5
L-Do_31_Mep_10_	70	14.4	10.5	1.15	n.d.	58
L-Ni_13_Mo_4_	70	12.8	11.9	1.15	n.d.	94
L-Ni_13_Mep_4_	70	12.8	11.6	1.18	n.d.	65
L-Phe_13_Mo_4_	70	13.5	10.9	1.16	n.d.	92.5
L-Phe_13_Mep_4_	70	13.5	9.2	1.25	n.d.	81
L-Do_13_Mo_4_	70	14.4	9.0	1.32	n.d.	69.5
L-Do_13_Mep_4_	70	14.4	8.8	1.37	n.d.	78.5
H-Ni_31_Mo_10_	115	18.3	17.5	1.21	100	91.5
H-Ni_31_Mep_10_	115	18.3	19.1	1.15	93.6	85.5
H-Phe_31_Mo_10_	115	20.3	19.1	1.18	99.3	84.5
H-Phe_31_Mep_10_	115	20.3	16.7	1.15	100	92.5
H-Do_31_Mo_10_	115	23.6	16.6	1.27	100	95
H-Do_31_Mep_10_	115	23.7	15.6	1.24	96.7	81.5
L-Bam_31_Mep_10_	70	11.7	12.1	1.25	85.6	69
L-Bmam_31_Mep_10_	70	12.7	12.1	1.27	88	68
L-Tmb_31_Mep_10_	70	13.3	8.8	1.28	99.3	63
L-Oct_31_Mep_10_	70	13.3	10.4	1.22	99	51
L-Olam_31_Mep_10_	70	15.5	8.1	1.27	96.7	71
L-Do_30_Mep_5_	70	14.75	11.4	1.23	n.d.	59
L-Tmb_5_Mo_90_	70	10.16	10.6	1.21	n.d.	100
L-Oct_5_Mep_5_	70	14.07	12.5	1.20	n.d.	100
L-Phe_15_Mo_30_	70	12.06	12.1	1.18	n.d.	89

a Target DP and *M*_n_ are theoretical values.

b *M*_n_ and *Đ* were measured via GPC. *M*_n_ was determined by comparison to PEG standards, except for L-Tmb_5_Mo_90_, which was compared to PMMA standards.

c Percent conversion was determined via ^1^H NMR.

d Percent yield was determined by mass after lyophilization.

The copolymers were characterized by gel permeation chromatography (GPC; Figure S13). NaBF_4_ (1 wt %) was included in the mobile phase during GPC analysis to ensure the solubility of the polymers. The polymer GPC spectra were analyzed until the solvent elution time of 19.2 min.^72^ Several samples showed tailing on the refractive index, indicative of polymer interactions with the column, which is common for cationic polymers. Some of the samples, most notably those with a higher DP, showed a bimodal distribution; however, most samples were monomodal, as expected from RAFT polymerization. Molecular weights were determined by comparison to PEG standards, except for L-Tmb_5_Mo_90_. Because of this formulation’s low cationic density, it was more appropriately evaluated by comparison to PMMA standards. The dispersities are typical for controlled radical polymerization techniques (Table 1). On average, we observed a yield of 84%. Several of these polymers are highly hygroscopic and yields above 100% are observed for two polymers due to the presence of residual water.

### Antibacterial Efficacy and Safety of Polyacrylamide Copolymers

**Figure 3:**
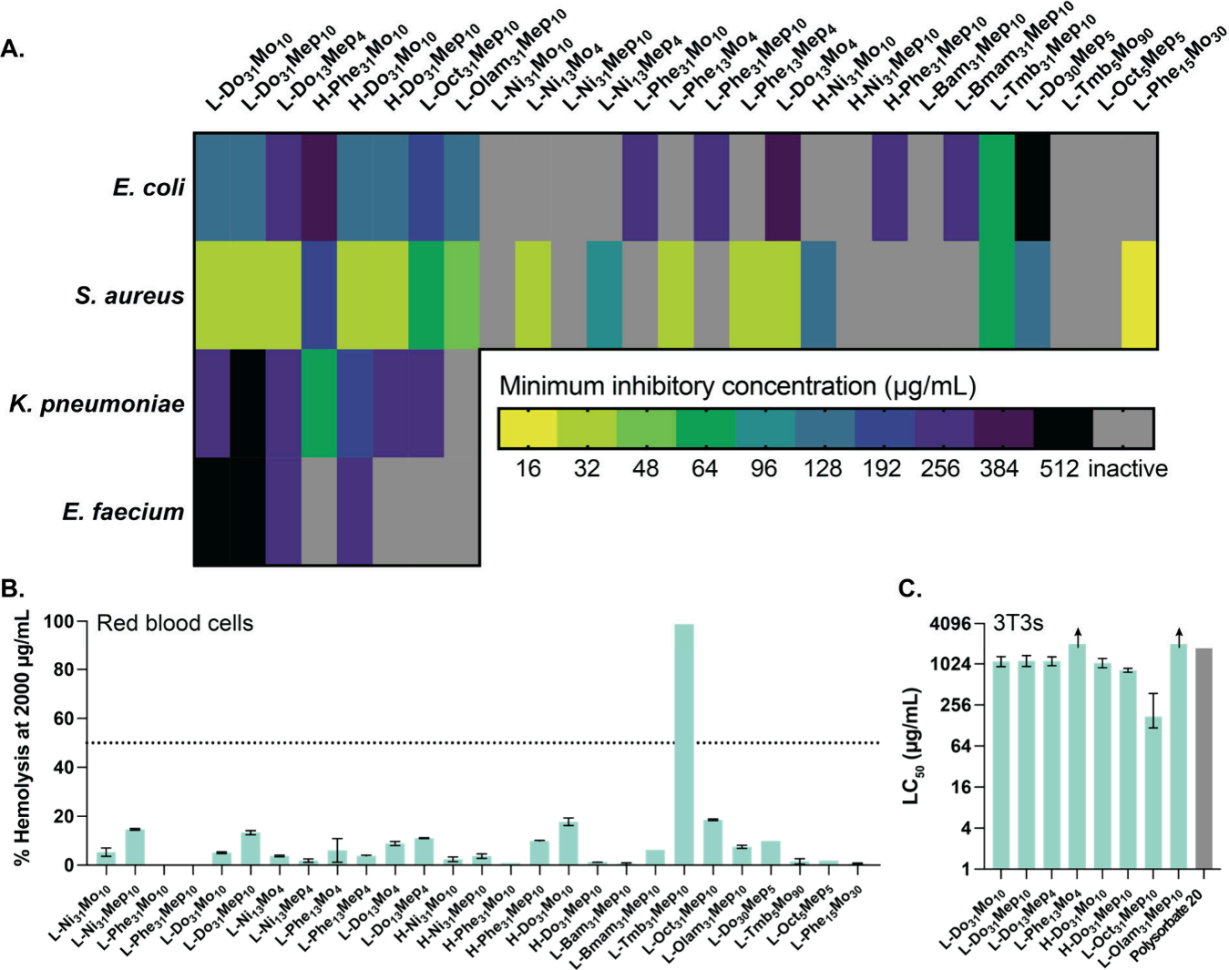
Efficacy and safety of novel polyacrylamides. (A) Heat map showing the antibacterial efficacy (MIC) of each polymer against several bacteria as measured after overnight inoculation. (B) Hemolytic activity of each polymer over 1 h at a concentration of 2000 μg/mL. (C) LC_50_ values of eight copolymers against 3T3 cells measured at 24 h and compared to a commercial excipient control, polysorbate 20.

To evaluate the efficacy of each novel copolymer, its minimum inhibitory concentration (MIC) was determined against a standard Gram-negative strain, *E. coli* ATCC 25922, and a standard Gram-positive strain, *S. aureus* ATCC 29213 (Figure 3A, Table 2). Of the 27 copolymers evaluated, 20 showed activity against at least one of these targets, and 11 showed activity against both, indicating broad-spectrum efficacy. We observed no trade-off between efficacy against *S. aureus* compared to *E. coli*, with several copolymers demonstrating robust activity against both (Figure S14). The observed MIC values were comparable to those of previously reported antimicrobial polymers and licensed antibiotic drug products (Table 2).^28−30,32,35,36,46,52,73−75^

**Table 2:** Antibacterial Efficacy of Copolyacrylamides and Penicillin G

	Hydrophobic/cationic molar ratio^a^	Hydrophobicity (∑LogP)^b^	MIC: *E. coli*, μg/mL (μM)^c^	MIC: *S. aureus*, μg/mL (μM)^c^	MIC: *K. pneumoniae*, μg/mL (μM)^c^	MIC: *E. faecium*, μg/mL (μM)^c^
Penicillin G	N/A	N/A	32 (89.8)	0.5–1 (1.40–2.81)	n.d.	n.d.
L-Ni_31_Mo_10_	0.95	0.80	>512 (>46.0)	>512 (>46.0)	n.d.	n.d.
L-Ni_31_Mep_10_	0.95	1.20	>512 (>43.1)	>512 (>43.1)	n.d.	n.d.
L-Phe_31_Mo_10_	0.73	1.70	256 (24.9)	>512 (>49.7)	n.d.	n.d.
L-Phe_31_Mep_10_	0.73	2.10	256 (24.3)	>512 (>48.7)	n.d.	n.d.
L-Do_31_Mo_10_	0.45	5.80	128 (12.3)	32–64 (3.07–6.14)	256 (24.5)	512 (49.1)
L-Do_31_Mep_10_	0.45	6.20	128 (10.5)	32 (2.64)	512 (42.2)	512 (42.2)
L-Ni_13_Mo_4_	0.29	0.80	>512 (>37.6)	32 (2.35)	n.d.	n.d.
L-Ni_13_Mep_4_	0.29	1.20	>512 (>37.5)	64–128 (4.69–9.39)	n.d.	n.d.
L-Phe_13_Mo_4_	0.22	1.70	>512 (>40.7)	32 (2.54)	n.d.	n.d.
L-Phe_13_Mep_4_	0.22	2.10	>512 (>44.4)	32 (2.78)	n.d.	n.d.
L-Do_13_Mo_4_	0.14	5.80	256–512 (21.5–43.1)	32 (2.69)	n.d.	n.d.
L-Do_13_Mep_4_	0.14	6.20	256 (21.2)	32 (2.64)	256 (21.2)	256 (21.2)
H-Ni_31_Mo_10_	0.95	0.80	>512 (>24.1)	128 (6.02)	n.d.	n.d.
H-Ni_31_Mep_10_	0.95	1.20	>512 (>23.3)	>512 (>23.3)	n.d.	n.d.
H-Phe_31_Mo_10_	0.73	1.70	256–512 (11.4–22.7)	128–256 (5.68–11.36)	64 (2.84)	>512 (>22.7)
H-Phe_31_Mep_10_	0.73	2.10	256 (13.4)	>512 (>26.8)	n.d.	n.d.
H-Do_31_Mo_10_	0.45	5.80	128 (6.10)	32 (1.52)	128–256 (6.10–12.2)	256 (12.2)
H-Do_31_Mep_10_	0.45	6.20	128 (6.65)	32 (1.66)	256 (13.3)	>512 (>26.6)
L-Bam_31_Mep_10_	0.84	1.50	>512 (>33.8)	>512 (>33.8)	n.d.	n.d.
L-Bmam_31_Mep_10_	0.68	1.62	256 (16.6)	>512 (>33.3)	n.d.	n.d.
L-Tmb_31_Mep_10_	0.59	2.84	64 (5.67)	64 (5.67)	n.d.	n.d.
L-Oct_31_Mep_10_	0.59	3.17	128–256 (10.1–20.3)	64 (5.06)	256 (20.3)	>512 (>40.5)
L-Olam_31_Mep_10_	0.33	7.03	128 (12.4)	32–64 (3.11–6.22)	>512 (>49.8)	>512 (>49.8)
L-Do_30_Mep_5_	0.40	6.20	512 (36.6)	128 (9.14)	n.d.	n.d.
L-Tmb_5_Mo_90_	1.13	2.44	>512 (>39.9)	>512 (>39.9)	n.d.	n.d.
L-Oct_5_Mep_5_	0.06	3.17	>512 (>34.1)	>512 (>34.1)	n.d.	n.d.
L-Phe_15_Mo_30_	0.38	1.70	>512 (>36.0)	16 (1.13)	n.d.	n.d.

a The hydrophobic/cationic molar ratio is calculated from the monomer feed ratios.

b The hydrophobicity is computationally calculated using the predicted LogP of the hydrophobic and hydrophilic monomers using ChemDraw.

c MIC values were determined after overnight inoculation.

The hemolytic activity of each copolymer was measured as a metric of mammalian nontoxicity. Most copolymers showed remarkably low hemolytic activity, even up to concentrations exceeding 2000 μg/mL, indicating favorable safety profiles (Figure 3B, Table 3). Indeed, for most polymers, HC_50_ (the concentration at which 50% of red blood cells are lysed) values could not be determined because it fell beyond the concentration range tested. Even as research in the field has yielded polymers with similarly promising hemolysis profiles, these results remain among the best that have been reported in the literature.^22,46,64,65,75^ Our polymers demonstrated clear selectivity for permeabilizing bacteria, leaving red blood cells intact. Interestingly, the antibacterial efficacy of the copolymer did not directly correlate with the hemolytic activity. The promising safety profile of our copolymers demonstrates the importance of our ternary copolymer design, where the introduction of a hydrophilic monomer enabled access to copolymers with potent antibacterial activity and low toxicity, as expected.

**Table 3:** *In Vitro* Safety of Polyacrylamide Copolymers and Polysorbate 20

	HC_50_, μg/mL (90% CI)^a^	LC_50_, 3T3s, μg/mL (90% CI)^b^	LC_50_, A549s, μg/mL (90% CI)^b^
Polysorbate 20	n.d.	1800	1700 (200–2000)
L-Ni_31_Mo_10_	>2000	n.d.	n.d.
L-Ni_31_Mep_10_	>2000	n.d.	n.d.
L-Phe_31_Mo_10_	>2000	n.d.	n.d.
L-Phe_31_Mep_10_	>2000	n.d.	n.d.
L-Do_31_Mo_10_	>2000	1100 (900–1300)	200 (100–300)
L-Do_31_Mep_10_	>2000	1100 (900–1400)	80 (40–100)
L-Ni_13_Mo_4_	>2000	n.d.	n.d.
L-Ni_13_Mep_4_	>2000	n.d.	n.d.
L-Phe_13_Mo_4_	>2000	n.d.	n.d.
L-Phe_13_Mep_4_	>2000	n.d.	n.d.
L-Do_13_Mo_4_	<50	n.d.	n.d.
L-Do_13_Mep_4_	>2000	1100 (1000–1300)	60 (30–80)
H-Ni_31_Mo_10_	>8000	n.d.	n.d.
H-Ni_31_Mep_10_	>8000	n.d.	n.d.
H-Phe_31_Mo_10_	>8000	>2048	200 (90–300)
H-Phe_31_Mep_10_	>8000	n.d.	n.d.
H-Do_31_Mo_10_	>8000	1100 (900–1200)	100 (40–300)
H-Do_31_Mep_10_	6000 (5000–8000)	820 (770–880)	70 (30–110)
L-Bam_31_Mep_10_	>8000	n.d.	n.d.
L-Bmam_31_Mep_10_	6000 (5000–8000)	n.d.	n.d.
L-Tmb_31_Mep_10_	<62.5	n.d.	n.d.
L-Oct_31_Mep_10_	5000 (4000–6000)	200 (100–400)	40 (30–100)
L-Olam_31_Mep_10_	>8000	>2048	90 (70–100)
L-Do_30_Mep_5_	3000 (3000–4000)	n.d.	n.d.
L-Tmb_5_Mo_90_	>4000	n.d.	n.d.
L-Oct_5_Mep_5_	>4000	n.d.	n.d.
L-Phe_15_Mo_30_	>4000	n.d.	n.d.

a HC_50_ values were measured after a 1 h incubation with mammalian red blood cells.

b LC_50_ values were measured after 24 h.

Based on the results of our screen with *E. coli* and *S. aureus*, as well as the hemolysis profiles, eight copolymers were selected as top-performing candidates for further evaluation. These candidates were then tested against two additional bacteria: *K. pneumoniae* ATCC 13884 (Gram-negative) and *E. faecium* ATCC 35667 (Gram-positive). These bacterial species, along with *E. coli* and *S. aureus*, are members of a group of pathogens known for their ability to develop resistance to antibiotics.^76,77^ Each species is on the WHO priority list on account of its threat to global health.^78^ Among the eight copolymers tested, seven demonstrated activity against at least one additional strain, while four exhibited activity against both. The efficacy of our leading copolymers against these species represents a clear step toward combating antimicrobial resistance.

To further evaluate the safety of our leading copolymers, we examined the cytotoxicity of our top-performing candidates. We determined the LC_50_ following 24 h of exposure for 3T3 cells (Figure 3C, Table 3) and A549 cells (Figure S15, Table 3). The LC_50_ values were compared to those for polysorbate 20, one of a class of excipients used broadly in FDA-approved drug products. Notably, polysorbate 20 is approved for intranasal administration at a concentration of 25 mg/mL,^79^ more than an order of magnitude above its LC_50_ for either of the cell lines tested. Moreover, polysorbates are approved for intravenous administration at doses of ∼4,700 mg (∼78 mg/kg in humans), indicating that despite their observed LC_50_ values *in vitro*, polysorbates are well tolerated *in vivo*. Our copolymers exhibited comparable LC_50_ values in 3T3 cells but lower LC_50_ values in A549 cells than the polysorbate control. Given the relationship between observed LC_50_ values and tolerable dosing *in vivo* for polysorbates, these cytotoxicity studies suggest that the tolerability of our copolymers will be sufficiently high to provide a robust therapeutic margin for dosing *in vivo*. These results corroborate the safety of these antibiotic copolymer candidates in the hemolysis assays described above.

### Polyacrylamide Copolymer Antibiotics Function through Membrane Disruption

**Figure 4:**
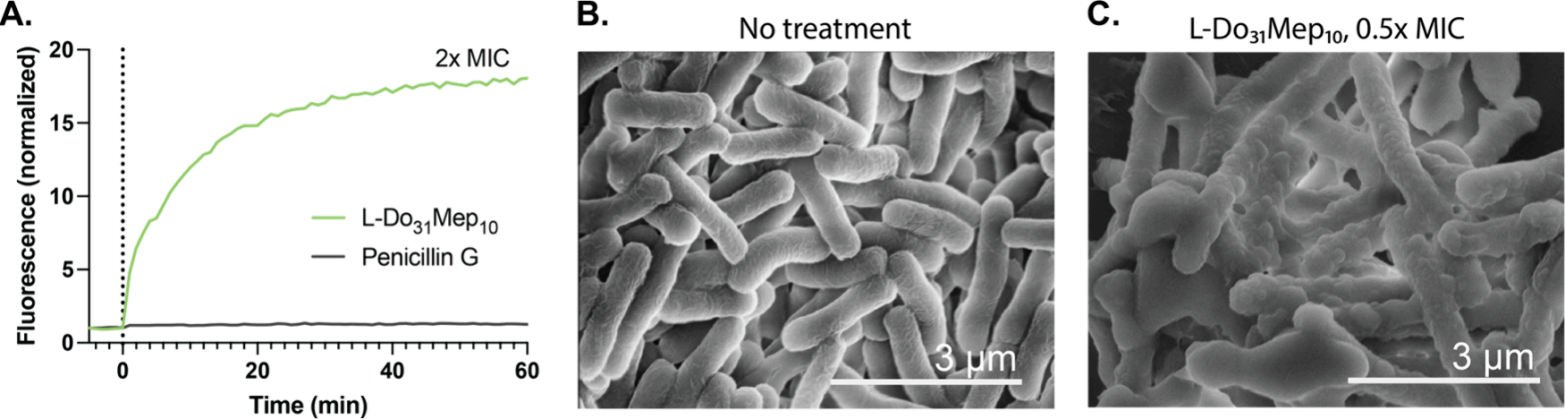
L-Do_31_Mep_10_ disrupts the membrane of *E. coli*. (A) Membrane permeabilization assay, using the fluorescent probe propidium iodide, monitored continuously by plate reader. (B) SEM image of *E. coli* (untreated). (C) SEM image of *E. coli* treated with L-Do_31_Mep_10_.

Based on the efficacy and safety data, we selected one top candidate copolymer, L-Do_31_Mep_10_, for further characterization. We sought to verify our hypothesized mechanism of cell-killing behavior through membrane disruption. We incubated *E. coli* with the DNA-intercalating dye propidium iodide in the presence of L-Do_31_Mep_10_. We observed a dramatic increase in fluorescence over an hour of monitoring (Figure 4A), indicating that both the outer and inner membranes of *E. coli* had been compromised, thereby allowing the propidium iodide to access the DNA within the bacteria. Our negative control, penicillin G, kills bacteria through inhibiting cell wall synthesis and did not lead to a similar increase in fluorescence.

Further, we visually examined *E. coli* before and after exposure to L-Do_31_Mep_10_ at to half the lethal level (0.5x MIC; 64 μg/mL; 6.27 μM) using scanning electron microscopy (SEM) (Figure 4B–C). Perturbations in the membrane were observed in the presence of L-Do_31_Mep_10_. Notably, blistering was visible in the treated sample, consistent with disruptions to the physical integrity of the membrane.

### Polyacrylamide Copolymer Antibiotics Mitigate the Onset of Resistance

**Figure 5:**
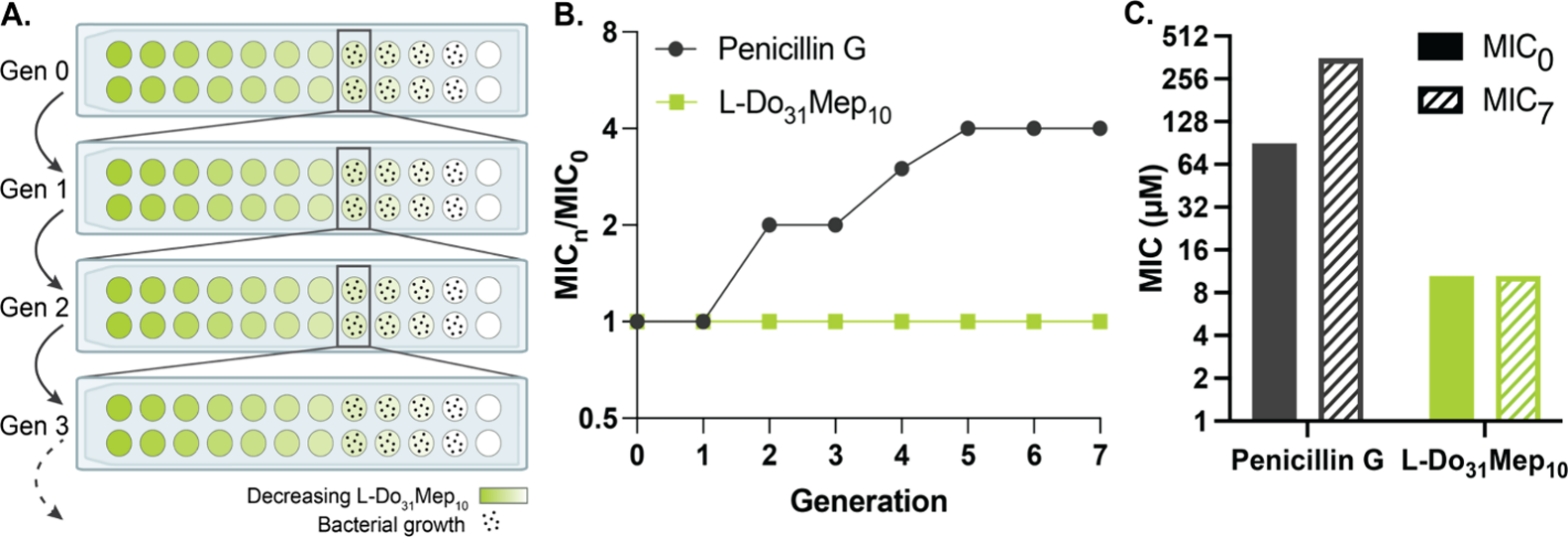
L-Do_31_Mep_10_ evades resistance mechanisms. (A) Schematic indicating the workflow for the resistance assay. (B) Results of resistance assay, indicating that L-Do_31_Mep_10_ is immune to resistance over 7 generations. (C) Comparison of MIC_0_ and MIC_7_ for penicillin G and L-Do_31_Mep_10_.

We hypothesized that a membrane disruption mechanism would allow our treatments to overcome traditional mechanisms of resistance. We tested this hypothesis by exposing *E. coli* ATCC 25922 to half the lethal level of L-Do_31_Mep_10_ for 7 passages. In each passage, we calculated the MIC of the bacteria (MIC_n_) and compared it to the MIC of naïve *E. coli* (MIC_0_). We compared the copolymer efficacy to a small molecule control (penicillin G). Penicillin G is not reliably effective against clinical *E. coli* isolates on account of widespread resistance developed over several decades of penicillin G overuse, and newer penicillin derivatives, including ampicillin and amoxicillin, are more commonly used to treat infections in patients.^80^ For these assays we selected a strain of *E. coli* with known susceptibility to penicillin G, in accordance with previous reports.^81,82^ In these studies, no resistance to the copolymer was observed over the course of the experiment, whereas resistance to penicillin G quickly developed over the same time frame (Figure 5). These results indicate that the physical membrane disruption mechanism of the copolymer can mitigate the development of resistance.

### Polyacrylamide Copolymer Antibiotics Can Rehabilitate Traditional Antibiotics

**Figure 6:**
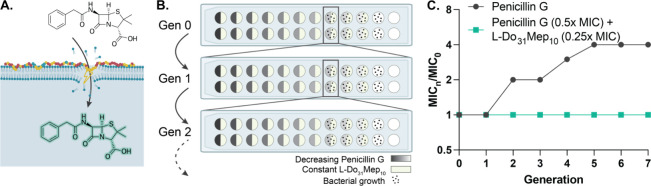
L-Do_31_Mep_10_ improves the efficacy of penicillin G. (A) Schematic showing the hypothesized mechanism of membrane perturbation that facilitates antibiotic uptake. (B). Schematic detailing the resistance assay where the copolymer is used as an adjuvant to penicillin G. (C) Results of resistance assay, indicating that combination treatment prevents resistance against penicillin G over 7 generations.

We sought to evaluate the potential of our leading polyacrylamide copolymer to improve the efficacy of existing antibiotics. Previous work demonstrated that polymers can improve the delivery and potency of small-molecule antibiotics.^11,13,53,83^ We hypothesized that, since our novel copolymer disrupts the membrane of bacteria, it could facilitate entry of antibiotics into the cell (Figure 6A). We evaluated a range of antibiotics, including one that is already effective against *E. coli* (rifampicin), one that is ineffective due to an inability to access to the periplasmic space (vancomycin), and one that shows moderate efficacy due to incomplete access to the periplasmic space (penicillin G).^84−86^ We determined the MIC of each antibiotic alone and in the presence of a sublethal amount (0.5x MIC) of L-Do_31_Mep_10_. Cotreatment decreased the MIC by a factor of 4 for penicillin G and by a factor of 2 for vancomycin and rifampicin, supporting our hypothesis that the copolymer may improve intracellular access for each of these antibiotics (Figure S16). Thus, clinically relevant antibiotics become more potent in combination treatment with the antibacterial polyacrylamide.

Additionally, we sought to determine our copolymer’s ability to affect the development of resistance to penicillin G. It has been previously demonstrated that a combination treatment of an antimicrobial polymer and a small-molecule antibiotic can mitigate the development of resistance, although this is likely due to the polymer’s ability to circumvent resistance, as the concentration of the polymer varied alongside the small molecule in this resistance assay.^62,87^ Another study demonstrated that a polymer could be used to protect a small-molecule antibiotic from the development of resistance; however, the polymer described had minimal antimicrobial activity on its own.^63^ We hypothesized that our antimicrobial polymers, in addition to showing efficacy in isolation, could also act as an adjuvant to protect a small-molecule antibiotic from resistance. To investigate this hypothesis, we repeated the resistance study, but we used L-Do_31_Mep_10_ as an adjuvant at a constant sublethal concentration (0.25x MIC; 32 μg/mL; 2.64 μM). We evaluated the development of resistance to penicillin G over 7 passages in the presence of L-Do_31_Mep_10_, and we observed no resistance (Figure 6B,C). This remarkable finding suggests that our leading copolymer antibiotic may serve as a tool to combat the development of resistance against small-molecule antibiotics that are subject to traditional resistance mechanisms on their own.

### Evaluation of the Physicochemical Properties Driving Efficacy of Polyacrylamide Copolymers

**Figure 7:**
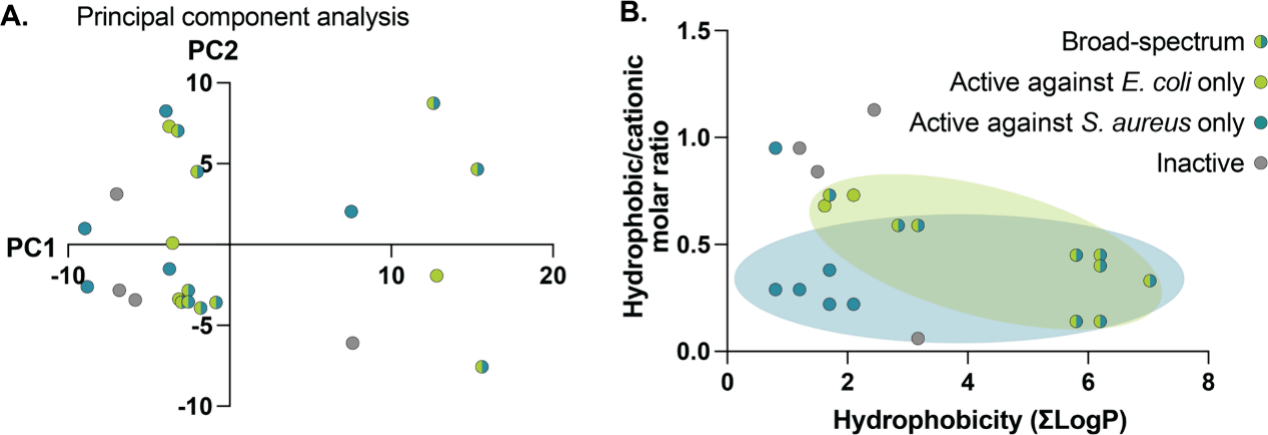
Evaluation of chemical differences in the library of novel polyacrylamides. Each point represents a polymer entry in our library, color-coded by its antimicrobial activity. (A) Principal component analysis was used to condense highly dimensional chemical data of each polymer, featurized using the RDKit package. (B) The impact of hydrophobicity and hydrophobic/cationic balance on the efficacy of novel copolymers. Molar ratios were calculated from monomer feed ratios, and hydrophobicity (LogP) was calculated for the hydrophobic and hydrophilic monomers using ChemDraw.

In addition to evaluation of the antibacterial efficacy and safety of our polyacrylamide copolymers, we analyzed each copolymer by some key physicochemical characteristics to investigate the influence of these properties on antibacterial efficacy. We featurized each copolymer using the RDKit package to compute hundreds of chemical characteristics.^88^ We condensed these multidimensional data using a principal component analysis (PCA), which preserved 60% of the variation of the data set (Figure 7A). In this analysis, we noticed little clustering, indicating that our effective copolymers were spread across the chemical space of our library. This observation likely arose because most of our entries were effective against at least one strain of bacteria. An expansion of the library in future studies might yield more robust patterns in the PCA.

We observed a strong relationship between the hydrophobicity of the monomers used and the antibacterial efficacy of the polymer. To explore this, we calculated the sum of the estimated LogP values (calculated by ChemDraw) for the hydrophobic and hydrophilic monomers used for each polymer (Table 2, Figure 7). We also investigated the influence of the molar ratio of the hydrophobic monomer to the cationic monomer (Table 2, Figure 7). We found that the copolymers with the great breadth of activity were those with the highest LogP values. In contrast, the least effective copolymers had low LogPs and high hydrophobic/cationic molar ratios, suggesting that these copolymers comprised a high concentration of insufficiently hydrophobic monomers. In this analysis, we also noticed that copolymers with a higher charge density showed preferential activity toward *S. aureus*, while those with a slightly higher hydrophobic/cationic ratio were relatively more active toward *E. coli*.

## Conclusion

We synthesized a library of 27 polyacrylamide copolymers at gram-scale with high yield using RAFT polymerization. We utilized statistical terpolymers to provide flexibility in independently varying the charge density and hydrophobicity of our library entries. We evaluated their activity as antibacterial agents against *E. coli*, *S. aureus*, *K. pneumoniae*, and *E. faecium* and found several copolymers that showed broad-spectrum activity. Further, we evaluated the safety of these copolymers *in vitro*, and we determined that they were highly selective for bacteria over mammalian cells, leaving the latter intact even at high concentrations. Some copolymers showed safety profiles comparable to a common excipient used in FDA-approved drug products.

One candidate copolymer, L-Do_31_Mep_10_, was selected for further characterization on account of its exceptional breadth and safety profile. Imaging studies confirmed that the copolymer functions through a membrane-disruption mechanism of cell-killing, which was found to both enhance the efficacy of traditional small-molecule antibiotics by improving access into the bacteria, as well as to mitigate traditional resistance mechanisms. Furthermore, a combination treatment of this leading copolymer with penicillin G prevented *E. coli* from developing resistance to the penicillin G over the course of the experiment, despite the copolymer being present well below its own lethal dose. To our knowledge, this is the first report of an antimicrobial copolymer protecting a small molecule from the development of resistance. This remarkable result suggests that these polyacrylamide copolymers may be useful as adjuvants to rehabilitate clinically used antibiotics in the fight against antimicrobial resistance.

## Supporting Information Available

Experimental methods and materials, NMR spectra of synthesized monomers, NMR and GPC spectra of polymers, compositional analysis of polymers, and additional efficacy and safety data (PDF)
Transparent Peer Review report available (PDF)
