# Supplementary material for: Polyacrylamide-Based Antimicrobial Copolymers to Replace or Rescue Antibiotics
Source: ACS Cent Sci. 2025 Mar 13;11(3):486–96. doi: 10.1021/acscentsci.4c01973 (PMC11950845; doi:10.1021/acscentsci.4c01973)
Supplement: Supplementary file 2 — oc4c01973_si_002.pdf [file oc4c01973_si_002.pdf]

oc-2024-01973k.R1

Name: Peer Review Information for "Polyacrylamide-based Antimicrobial Copolymers to Replace or Rescue Antibiotics"

First Round of Reviewer Comments

Reviewer: 1

Comments to the Author

This a very well written and interesting article which proposes the design of antimicrobial polymers to fight a significant upcoming problem is antibiotic resistance. The authors have prepared library of antimicrobial polymers using acrylamide monomers. By adjusting the type of hydrophobic monomers, the authors optimzed their antimicrobial activity. The paper is interesting and i recommend publication. However, the authors should cite recent works from Boyer's group, including Nature Communications 15 (1), 6818 (used of polyacrylamides as antimicrobial agents), Biomacromolecules 21 (12), 5241-5255 (effect of hydrophobic group on antimicrobial polymers).

Comments:

1. why the yeild can be higher than 100% (see table 1, first rows)

2. page 8, the incorporation of hydrophilic monomer to regulate biocompatibility is interesting and has been reported see: Effect of hydrophilic groups on the bioactivity of antimicrobial polymers; i recommend the inclusion of this paper and mention of this paper.

The work on the evolution study is interesting, but this paper should be cited: ACS infectious diseases 5 (8), 1357-1365. in this paper, the authors showed that bacteria do not become resistant to polymers. Furthermore, the polymers can restore the efficiency of antibiotic.

SI: the section is relatively short and I think the authors should provide more information on the caption of some figures, such as GPC (Figure S4). NMR and GPC methods should be separated from the polymerization to allow the readers to find them. I also have a comment on GPC, the authors should comment on the solubility of these polymers in DMF. The cationic charge may disturb their analyses.

Reviewer: 2

Comments to the Author

The manuscript of the Appel group reports on a series of ternary copolyacrylamides with cationic, hydrophobic, and hydrophobic moieties prepared by RAFT polymerization. Some of them show good antibacterial properties and low toxicity towards at least 3T3 cells. The synergistic effect with antibiotics is remarkable and also the lower onset of resistance formation as well. Altogether, the study is nicely following previous work of the group. However, the manuscript is hard to read due to the confusing abbreviations and missing details that are found to some extend in the supporting information. Further, the manuscript contains numerous overclaims regarding novelty of the findings and interpretation of the data. Also the exact composition of the polymers is never mentioned, because the cationic parts are not mentioned at all. Therefore, the manuscript needs revision as mentioned in the following:

Statistic copolymerization does not lead to homogeneous distribution of the monomers in the polymer. Particularly controlled of living polymerization often leads to gradient polymers depending on the copolymerization parameters. Therefore, it is important to discuss copolymerization parameters to judge a possible structure. The dispersity values suggest that the polymerization was not performed to full conversion of all monomers, but was stopped at a certain point. Full conversion with RAFT never leads to lowly dispersed polymers. Please describe the experimental procedure of the copolymerization in more detail with respect to truly used monomer composition and conversion or at least yield.

What are the real compositions of the polymers? Table 1 should contain the targeted composition of all three monomers and the composition found by 1HNMR. Why is Mw targeted. Monomer/RAFT ratio targets Mn. Supporting Figure S3, which seems to be a 1HNMR, suggests that the major part of the polymer is the cationic monomer. What is x in this formula?

Figure S4 shows that the SEC traces of the polymers are not monomodal. What are the smaller peaks and which part is used to calculate dispersity?

There are numerous cationic, more active antimicrobial polymers in the literature that do not lyse blood cells. This should be addressed.

In general, all figure and table captions should contain experimental details; otherwise the manuscript is very hard to read. Table 3 should contain the data of LC50 of A549s. Given the data in Figure S5 they are rather toxic. Considering that not LC50, but the concentration for survival of 90 or at least 80 % of the mammalian cells is the relevant concentration to judge cytotoxicity, it can clearly be stated that the antibacterially active polymers are rather toxic at least to A549s. The discussion is misleading and is not supported by the data. This should be corrected.

Cationic polymers including polypeptides are not forming resistance in bacteria in general. That is not new and should not be stated as such.

Testing resistance formation for only 7 days does not allow to judge the term that no resistance is formed. There are many test in the literature that are performed for 21 days or longer and even those do not always claim that no resistance will be formed, but the resistance formation is delayed. Please rephrase. Otherwise it sounds a bit over claimed.

The work of Hedrick and Yang is completely ignored. They have shown that cationic polymers supress resistance formation against various antibiotics. They have further shown that antibiotics can be transported into cells by cationic polymers, etc (e.g. https://doi.org/10.1002/adma.201302952). The statements that such things are observed for the first time are wrong and should be removed and discussed in the light of the literature.

Author's Response to Peer Review Comments:

Reviewer 1:

This a very well written and interesting article which proposes the design of antimicrobial polymers to fight a significant upcoming problem is antibiotic resistance. The authors have prepared library of antimicrobial polymers using acrylamide monomers. By adjusting the type of hydrophobic monomers, the authors optimzed their antimicrobial activity. The paper is interesting and i recommend publication. However, the authors should cite recent works from Boyer's group, including Nature Communications 15 (1), 6818 (used of polyacrylamides as antimicrobial agents), Biomacromolecules 21 (12), 5241-5255 (effect of hydrophobic group on antimicrobial polymers).

We thank the reviewer for this suggestion. These citations have been added as references 49 and 64 in our revised manuscript. In addition, we list here several other citations for relevant works from Boyer's group that we have included in our manuscript:

[22] Pham, P.; Oliver, S.; Boyer, C. Design of Antimicrobial Polymers. *Macromol. Chem. Phys*. **2023**, *224* (3), 2200226. https://doi.org/10.1002/macp.202200226.

[30] Pham, P.; Oliver, S.; Nguyen, D. T.; Boyer, C. Effect of Cationic Groups on the Selectivity of Ternary Antimicrobial Polymers. *Macromol. Rapid Commun*. **2022**, *43* (21), 2200377. https://doi.org/10.1002/marc.202200377.

[46] Nguyen, T.-K.; Lam, S. J.; Ho, K. K. K.; Kumar, N.; Qiao, G. G.; Egan, S.; Boyer, C.; Wong, E. H. H. Rational Design of Single-Chain Polymeric Nanoparticles That Kill Planktonic and Biofilm Bacteria. *ACS Infect. Dis*. **2017**, *3* (3), 237–248. https://doi.org/10.1021/acsinfecdis.6b00203.

[53] Jung, K.; Corrigan, N.; Wong, E. H. H.; Boyer, C. Bioactive Synthetic Polymers. *Adv. Mater.* **2022**, *34* (2), 2105063. https://doi.org/10.1002/adma.202105063.

[71] Judzewitsch, P. R.; Nguyen, T.-K.; Shanmugam, S.; Wong, E. H. H.; Boyer, C. Towards Sequence-Controlled Antimicrobial Polymers: Effect of Polymer Block Order on Antimicrobial Activity. *Angew. Chem. Int. Ed.* **2018**, *57* (17), 4559–4564. https://doi.org/10.1002/anie.201713036.

[72] Judzewitsch, P. R.; Zhao, L.; Wong, E. H. H.; Boyer, C. High-Throughput Synthesis of Antimicrobial Copolymers and Rapid Evaluation of Their Bioactivity. *Macromolecules* **2019**, *52* (11), 3975–3986. https://doi.org/10.1021/acs.macromol.9b00290.

[73] Namivandi-Zangeneh, R.; Kwan, R. J.; Nguyen, T.-K.; Yeow, J.; Byrne, F. L.; Oehlers, S. H.; Wong, E. H. H.; Boyer, C. The Effects of Polymer Topology and Chain Length on the Antimicrobial Activity and Hemocompatibility of Amphiphilic Ternary Copolymers. *Polym. Chem.* **2018**, *9* (13), 1735–1744. https://doi.org/10.1039/C7PY01069A.

[85] Namivandi-Zangeneh, R.; Sadrearhami, Z.; Dutta, D.; Willcox, M.; Wong, E. H. H.; Boyer, C. Synergy between Synthetic Antimicrobial Polymer and Antibiotics: A Promising Platform To Combat Multidrug-Resistant Bacteria. *ACS Infect. Dis.* **2019**, *5* (8), 1357–1365. https://doi.org/10.1021/acsinfecdis.9b00049.

1. why the yeild can be higher than 100% (see table 1, first rows)

We thank the reviewer for their attentive review of our manuscript. Several of our polymers are highly hydroscopic and so our observed yields exceed 100% because of residual water in these materials. To clarify for the reader, we have included the following explanation for this observation in our revised manuscript text:

Pages 6-7: "On average, we observed a yield of 84%. Several of these polymers are highly hygroscopic and yields above 100% are observed for two polymers due to the presence of residual water."

2. page 8, the incorporation of hydrophilic monomer to regulate biocompatibility is interesting and has been reported see: Effect of hydrophilic groups on the bioactivity of antimicrobial polymers; i recommend the inclusion of this paper and mention of this paper.

The work on the evolution study is interesting, but this paper should be cited: ACS infectious diseases 5 (8), 1357-1365. in this paper, the authors showed that bacteria do not become resistant to polymers. Furthermore, the polymers can restore the efficiency of antibiotic.

We thank the reviewer for this insightful suggestion. In our description of our library design, we have added a comment on the previously demonstrated impact of hydrophilicity on biocompatibility and included the recommended citation (68). In the description of our library results, we have added additional context to our observations (reproduced below).

In addition, our discussion of polymers used to adjuvant existing antibiotics has been modified to include the mentioned citation (85). That work is distinct from ours, in that it varied the concentration of polymer in the treatment group, while ours uses the polymer as an adjuvant and only varies the concentration of penicillin G in the assay.

Page 4: "Inclusion of a third monomer allowed for greater control in exploring the independent effects of charge and hydrophobicity (Figure 1E). It also allowed for control of hydrophilicity, which is known to impact antimicrobial activity and biocompatibility.[22,68]"

Page 8: "The promising safety profile of our copolymers demonstrates the importance of our ternary copolymer design, where the introduction of a hydrophilic monomer enabled access to copolymers with potent antibacterial activity and low toxicity, as expected."

SI: the section is relatively short and I think the authors should provide more information on the caption of some figures, such as GPC (Figure S4). NMR and GPC methods should be separated from the polymerization to allow the readers to find them. I also have a comment on GPC, the authors should comment on the solubility of these polymers in DMF. The cationic charge may disturb their analyses.

We have added a comment on the solubility of the polymers during GPC analysis to the main text of our revised manuscript. The NMR and GPC methods have been moved into a separate section in the methods section of the SI, and additional information regarding solvent has been added to the caption of the GPC figure in the SI. We've also included additional information in the SI regarding polymer composition and corresponding compositional variance. These additional texts are reproduced here:

Page 6: "$NaBF_4$ (1 wt%) was included in the mobile phase during GPC analysis to ensure the solubility of the polymers."

SI, page 3: "*Polymer characterization*
NMR spectra were recorded on a Bruker 500 MHz spectrometer. M_n, M_w, and dispersity values were determined via GPC implementing PEG or PMMA standards after passing through an SEC column (Resolve Mixed Bed Low divinylbenzene (DVB) (Jordi Labs)) in a mobile phase of DMF with 1 wt% $NaBF_4$ at 50 °C and a flow rate of 1.0 mL/min (Dionex UltiMate 3000 pump, degasser, and auto-sampler (Thermo Fisher Scientific))."

SI, page 9: "Table S1. Observed composition of polymers calculated by ^{1}H NMR."

SI, page 10: "**Figure S4. Compositional variance of L-Do$_{31}$Mep$_{10}$ and H-Do$_{31}$Mep$_{10}$.** Comparison between the compositional variance of two polymers that vary only by degree of polymerization (DP). Random incorporation of monomers was assumed, and predictions were made using the compositional drift program. **A.** For each of the predicted 1500 polymer chains, the molar ratio of each monomer is represented by a dash. Boxes are drawn to highlight values between the 5th and 95th percentile. **B.** Coefficient of variance for the molar ratio of each monomer class. These results demonstrate that the larger (higher DP) polymer shows lower compositional variance."

SI, page 10: "**Figure S5. Compositional variance of L-Do$_{31}$Mep$_{10}$ and L-Do$_{13}$Mep$_4$.** Comparison between the compositional variance of two polymers that vary only by monomer feed ratio. Random incorporation of monomers was assumed, and predictions were made using the compositional drift program. **A.** For each of the predicted 1500 polymer chains, the molar ratio of each monomer is represented by a dash. Boxes are drawn to highlight values between the 5th and

95th percentile. **B**. Coefficient of variance for the molar ratio of each monomer class. These results demonstrate that the monomer feed ratio affects the variance, where lower variance is observed in the predominant monomer as higher variance is observed in the monomers with lower feed ratios."

SI, page 11: "**Figure S6. GPC chromatograms** of each polymer (**A-E**) in DMF with 1 wt% $NaBF_4$. **F.** GPC chromatogram of a solvent control with no polymer. All spectra are normalized

and baseline-corrected."

Reviewer 2:

The manuscript of the Appel group reports on a series of ternary copolyacrylamides with cationic, hydrophobic, and hydrophobic moieties prepared by RAFT polymerization. Some of them show good antibacterial properties and low toxicity towards at least 3T3 cells. The synergistic effect with antibiotics is remarkable and also the lower onset of resistance formation as well. Altogether, the study is nicely following previous work of the group. However, the manuscript is hard to read due to the confusing abbreviations and missing details that are found to some extend in the supporting information. Further, the manuscript contains numerous overclaims regarding novelty of the findings and interpretation of the data. Also the exact composition of the polymers is never mentioned, because the cationic parts are not mentioned at all. Therefore, the manuscript needs revision as mentioned in the following:

We thank the reviewer for their supportive comments regarding the main outcomes of our research, namely the adjuvanting effect with antibiotics and lower onset of resistance formation, as well as their feedback. We have endeavored to address each of the reviewer's comments in detail below. We believe the central conclusions of our manuscript have been significantly strengthened and are now more impactful.

Statistic copolymerization does not lead to homogeneous distribution of the monomers in the polymer. Particularly controlled of living polymerization often leads to gradient polymers depending on the copolymerization parameters. Therefore, it is important to discuss copolymerization parameters to judge a possible structure. The dispersity values suggest that the polymerization was not performed to full conversion of all monomers, but was stopped at a certain point. Full conversion with RAFT never leads to lowly dispersed polymers. Please describe the experimental procedure of the copolymerization in more detail with respect to truly used monomer composition and conversion or at least yield.

We thank the reviewer for their rigorous attention to detail. The identity of the cationic monomer has been included (page 4), and it's structure can be seen in Figure 1. The yield of each polymer and is included in Table 1, and we comment on the average yield in the main text (page 6). Conversion data was determined for a subset of polymers and typically exceeded 95%, and these values are included in Table 1. For the same subset of polymers, the final composition, as observed by ^{1}H NMR, is presented in Table S1.

In addition, we calculated predicted composition for 1500 sample polymer chains from a given polymerization using the Composition Drift analysis originally developed by Prof. Ting Xu and colleagues to draw comparisons in compositional variance between chains within a polymer sample. We compared two samples that varied by degree of polymerization, and another set of samples that varied by monomer feed ratio, and we determined that higher DP and higher monomer feed ratio led to reduced compositional variance for a given monomer. This data is displayed in Figures S4 and S5.

Page 4: "Each polymer contained the same cationic monomer, (3-acrylamidopropyl)trimethylammonium chloride (Tma)."

Page 6: "The copolymers were analyzed by nuclear magnetic resonance (NMR; Figure S3). For several polymers, their conversion and composition were recorded (Table 1, Table S1). The observed compositions closely matched the target molar ratios of monomers."

Page 6: "On average, we observed a yield of 84%."

Page 6: "A computational analysis, using the previously reported program Compositional Drift, was performed to analyze compositional variance for a subset of polymers (Figures S4, S5).[69-71]"

SI, page 9: "Table S1. Observed composition of polymers determined by ^{1}H NMR."

SI, page 10: "**Figure S4. Compositional variance of L-$Do_{31}Mep_{10}$ and H-$Do_{31}Mep_{10}$.** Comparison between the compositional variance of two polymers that vary only by degree of polymerization (DP). Random incorporation of monomers was assumed, and predictions were made using the compositional drift program. **A.** For each of the predicted 1500 polymer chains, the molar ratio of each monomer is represented by a dash. Boxes are drawn to highlight values between the 5th and 95th percentile. **B.** Coefficient of variance for the molar ratio of each monomer class. These results demonstrate that the larger (higher DP) polymer shows lower compositional variance."

SI, page 10: "**Figure S5. Compositional variance of L-$Do_{31}Mep_{10}$ and L-$Do_{13}Mep_{4}$.** Comparison between the compositional variance of two polymers that vary only by monomer feed ratio. Random incorporation of monomers was assumed, and predictions were made using the compositional drift program. **A**. For each of the predicted 1500 polymer chains, the molar ratio of each monomer is represented by a dash. Boxes are drawn to highlight values between the 5th and 95th percentile. **B**. Coefficient of variance for the molar ratio of each monomer class. These results demonstrate that the monomer feed ratio affects the variance, where lower variance is observed in the predominant monomer as higher variance is observed in the monomers with lower feed ratios."

What are the real compositions of the polymers? Table 1 should contain the targeted composition of all three monomers and the composition found by 1HNMR. Why is Mw targeted. Monomer/RAFT ratio targets Mn. Supporting Figure S3, which seems to be a 1HNMR, suggests that the major part of the polymer is the cationic monomer. What is x in this formula?

We thank the reviewer for this comment. Compositional analysis was calculated for a subset of polymers and has been included in Table S1. The observed compositions closely match the feed ratios, demonstrating that the monomers were incorporated proportionally at the bulk scale. As mentioned above, to investigate the compositional variance between chains in a polymer sample we simulated 1500 random chains from a given polymerization using the Compositional Drift program. We compared polymer samples that varied both by DP (Figure S4) and monomer feed ratio (Figure S5). We determined that the polymer DPs used in all our experiments reported here are sufficiently high to ensure low compositional dispersity, corroborating our previous work (Prossnitz et al., Adv. Sci. 2025, 2409604), and that higher monomer feed ratios yield lower relative variance for a given monomer set.

In addition, we have revised Table 1 to show target M_n and measured M_n instead of M_w. We have added a clarifying statement explaining the naming scheme, so the target composition of each polymer is contained within the name (reproduced below).

Page 6: "Each polymer is named by its target degree of polymerization (H for a high DP of 115; L for a low DP of 70), and the identity and target weight percent of its hydrophobic and hydrophilic monomers. The remaining weight percent is accounted for by the cationic monomer, which is (3acrylamidopropyl)trimethylammonium chloride) in all cases. For example, L-$Ni_{31}Mo_{10}$ has a low degree of polymerization (70) and consists of (3-acrylamidopropyl)trimethylammonium chloride (59%), N-isopropylacrylamide (31%), and 4-acryloylmorpholine (10%)."

SI, page 9: "Table S1. Observed composition of polymers determined by ^{1}H NMR."

SI, page 10: "**Figure S4. Compositional variance of L-$Do_{31}Mep_{10}$ and H-$Do_{31}Mep_{10}$.** Comparison between the compositional variance of two polymer samples with distinct degrees of polymerization (DP). Random incorporation of monomers was assumed, and predictions were made using the compositional drift program. **A.** For each of the predicted 1500 polymer chains, the molar ratio of each monomer is represented by a dash. Boxes are drawn to highlight values between the 5th and 95th percentile. **B.** Coefficient of variance for the molar ratio of each monomer class. These results demonstrate that the larger (higher DP) polymer shows lower compositional variance."

SI, page 10: "**Figure S5. Compositional variance of L-$Do_{31}Mep_{10}$ and L-$Do_{13}Mep_4$.**

Comparison between the compositional variance of two polymer samples with distinct monomer feed ratios. Random incorporation of monomers was assumed, and predictions were made using the compositional drift program. **A**. For each of the predicted 1500 polymer chains, the molar ratio of each monomer is represented by a dash. Boxes are drawn to highlight values between the 5th and 95th percentile. **B**. Coefficient of variance for the molar ratio of each monomer class. These results demonstrate that the monomer feed ratio affects the variance, where lower variance is observed in the predominant monomer as higher variance is observed in the monomers with lower feed ratios."

Figure S4 shows that the SEC traces of the polymers are not monomodal. What are the smaller peaks and which part is used to calculate dispersity?

We thank the reviewer for their attention to detail and for recognizing our oversight. In order to investigate this question, we examined the chromatogram of a solvent control (Figure S6F). We determined that the elution time of our column is 19.2 minutes. We adjusted the polymer spectra (Figure S6A-E) to end at 19.2 minutes, as any signal after this time is an artifact of the measurement. Several of the polymers show "dragging," or a long tail at the low molecular weight side of the peak, which is characteristic of polymer interactions with the column and is common for cationic polymers. Few of our polymers (L-$Ni_{13}Mep_4$, H-$Ni_{31}Mep_{10}$, H-$Phe_{31}Mo_{10}$, H$Do_{31}Mo_{10}$, H-$Do_{31}Mep_{10}$) show significant low molecular weight shoulders, indicating a bimodal distribution. All other polymers evaluated appear monomodal. We have clarified this for the reader:

Page 6: "The polymer GPC spectra were analyzed until the solvent elution time of 19.2 minutes.[71] Several samples showed tailing on the refractive index, indicative of polymer interactions with the column, which is common for cationic polymers. Some of the samples, most notably those with a higher DP, showed a bimodal distribution; however, most samples were monomodal, as expected from RAFT polymerization. Molecular weights were determined by comparison to PEG standards, except for L-Tmb_5Mo_{90}. Because of this formulation's low cationic density, it was more appropriately evaluated by comparison to PMMA standards. The dispersities are typical for controlled radical polymerization techniques (Table 1)."

SI, page 11: "**Figure S6. GPC chromatograms** of each polymer (**A-E**) in DMF with 1 wt% $NaBF_4$. **F.** GPC chromatogram of a solvent control with no polymer. All spectra are normalized and baseline-corrected."

There are numerous cationic, more active antimicrobial polymers in the literature that do not lyse blood cells. This should be addressed.

We thank the reviewer for this comment, and we have clarified our findings in the context of the literature. We include mention of and citations for several works that report favorable hemolysis data. Our updated manuscript text is reproduced below.

Page 4: "Inclusion of a third monomer allowed for greater control in exploring the independent effects of charge and hydrophobicity. It also allowed for control of hydrophilicity, which is known to impact antimicrobial activity and biocompatibility.[22,68]"

Page 8: "Even as research in the field has yielded polymers with similarly promising hemolysis profiles, these results remain among the best that have been reported in the literature.[22,46,64,65,75]"

In general, all figure and table captions should contain experimental details; otherwise the manuscript is very hard to read. Table 3 should contain the data of LC_{50} of A549s. Given the data in Figure S5 they are rather toxic. Considering that not LC_{50}, but the concentration for survival of 90 or at least 80 % of the mammalian cells is the relevant concentration to judge cytotoxicity, it can clearly be stated that the antibacterially active polymers are rather toxic at least to A549s. The discussion is misleading and is not supported by the data. This should be corrected.

We thank the reviewer for their careful attention to our experimental details. We have adjusted our table and figure captions to include additional information. The A549 data is included in Table 3, and we contextualize the results of that assay in reference to the polysorbate control. While polysorbates exhibit some cytotoxicity with A549 and 3T3 cells *in vitro*, they are remarkably well tolerated at very high concentrations and doses *in vivo* in commercial drug products. Indeed, there are drug products approved for intranasal administration that comprise polysorbates at 25 mg/mL (i.e., more than an order of magnitude above the LC_{50} values determined here) as well as drug products approved for intravenous delivery at polysorbate doses up to ~4700 mg daily (i.e., ~78 mg/kg doses that are more than an order of magnitude higher than the highest antibiotic doses). Since our polymers show only slightly higher cytotoxicity *in vitro* than the polysorbate control, the tolerated dose *in vivo* is expected to be sufficiently high to provide a robust therapeutic margin for dosing. We have endeavored to clarify our rationale for the reader:

Page 11: "We determined the LC_{50} following 24 h of exposure for 3T3 cells (Figure 2C, Table 3) and A549 cells (Figure S6, Table 3). The LC_{50} values were compared to those for polysorbate 20, one of a class of closely related excipients used broadly in FDA-approved drug products. Notably, polysorbate 20 is approved for intranasal administration at a concentration of 25 mg/mL,[79] more than an order of magnitude above its LC_{50} for either of the cell lines tested. Moreover, polysorbates are approved for intravenous administration at doses of ~4,700 mg (~78 mg/kg in humans), indicating that despite their observed LC_{50} values *in vitro* polysorbates are well tolerated *in vivo*. Our copolymers exhibited comparable LC_{50} values in 3T3 cells but lower LC_{50} values in A549 cells than the polysorbate control. Given the relationship between observed LC_{50} values and tolerable dosing *in vivo* for polysorbates, these cytotoxicity studies suggest that the tolerability of our copolymers will be sufficiently high to provide a robust therapeutic margin for dosing *in vivo*. These results corroborate the safety of these antibiotic copolymer candidates in the hemolysis assays described above."

Page 5: “**Figure 1. Novel polymers to combat antimicrobial resistance. A.** Graph comparing the decreasing research into antibiotics with the increasing incidence of MDR bacteria. Values determined using NCBI tools: PubMed by Year and MicroBIGG-E. **B.** Schematic showing common mechanisms of AMR and the hypothesized ability of copolymers to overcome these mechanisms. **C.** Schematic of statistical copolymer library showing monomers used and their classes. **D.** RAFT polymerization conditions. **E.** The use of a ternary system allows for independent tuning of charge and hydrophobicity.”

Page 7: “**Table 1. Composition of polyacrylamide library**. Target DP and M_n are theoretical values. M_n and Đ were measured via GPC. Percent conversion was determined via NMR.”

Page 9: “**Figure 2. Efficacy and safety of novel polyacrylamides. A.** Heat map showing the antibacterial efficacy (MIC) of each polymer against several bacteria as measured after overnight inoculation. **B.** Hemolytic activity of each polymer over one hour at a concentration of 2000 μg/mL. **C.** LC_{50} values of eight copolymers against 3T3 cells measured at 24 hours and compared to a commercial excipient control, polysorbate 20.”

Page 9: “**Table 2. Antibacterial efficacy of novel polyacrylamides and penicillin G.** The hydrophobic/cationic molar ratio is calculated from the monomer feed ratios, and the hydrophobicity is computationally calculated using the predicted LogP of the hydrophobic and hydrophilic monomers using ChemDraw. The MIC values were determined after overnight inoculation.”

Page 11: “**Table 3. *In vitro* safety of polyacrylamide copolymers and polysorbate 20.** HC_{50} values were measured after a one hour incubation with mammalian red blood cells. LC_{50} values were measured after 24 hours.”

Page 13: “**Figure 3. L-$Do_{31}Mep_{10}$ disrupts the membrane of *E. coli*. A.** Membrane permeabilization assay, using the fluorescent probe propidium iodide, monitored continuously by plate reader. **B.** SEM image of *E. coli* (untreated). **C.** SEM image of *E. coli* treated with L$Do_{31}Mep_{10}$.”

Page 17: “**Figure 6. Evaluation of chemical differences in the library of novel polyacrylamides.** Each point represents a polymer entry in our library, color-coded by its antimicrobial activity. **A.** Principal component analysis was used to condense highly dimensional chemical data of each polymer, featurized using the ChemoPy package. **B.** The impact of hydrophobicity and hydrophobic/cationic balance on the efficacy of novel copolymers. Molar ratios were calculated from monomer feed ratios, and hydrophobicity (LogP) was calculated for the hydrophobic and hydrophilic monomers using ChemDraw.”

Cationic polymers including polypeptides are not forming resistance in bacteria in general. That is not new and should not be stated as such.

We thank the reviewer for this comment. We have now included in our manuscript mention of several previous works that have developed cationic antimicrobial polymers that have demonstrated reduced resistance formation.

Page 3: "Previous work has explored the development of antimicrobial polymers and oligomers, both as coatings for devices or surfaces[10–18] and as treatments.[19–49] These studies have identified positive charge and hydrophobicity as key parameters to enable antibacterial activity, and several of these polymers were shown to work through a membrane disruption mechanism.[50–64] This mode of action overcomes traditional resistance mechanisms, since the polymers don't need access to the intracellular space, and they aren't specific to a singular molecular target where mutations confer resistance.[65]"

Testing resistance formation for only 7 days does not allow to judge the term that no resistance is formed. There are many test in the literature that are performed for 21 days or longer and even those do not always claim that no resistance will be formed, but the resistance formation is delayed. Please rephrase. Otherwise it sounds a bit over claimed.

We have adjusted our language, so we do not say it "prevents the development of resistance." Instead, we note that it "prevents the development of resistance over the course of the experiment" (page 14, page 18) or that the polymers "can prevent or delay the onset of resistance" (page 28).

The work of Hedrick and Yang is completely ignored. They have shown that cationic polymers supress resistance formation against various antibiotics. They have further shown that antibiotics can be transported into cells by cationic polymers, etc (e.g. https://doi.org/10.1002/adma.201302952). The statements that such things are observed for the first time are wrong and should be removed and discussed in the light of the literature.

We thank the reviewer for this suggestion. This citation has been added (81).

Page 14: "Previous work has demonstrated that polymers can improve the delivery and potency of small-molecule antibiotics.[11,13,53,83]"

In addition, we list here several other citations for relevant works from collaborations between Hedrick's and Yang's groups that we have included:

[19] Engler, A. C.; Wiradharma, N.; Ong, Z. Y.; Coady, D. J.; Hedrick, J. L.; Yang, Y.-Y. Emerging Trends in Macromolecular Antimicrobials to Fight Multi-Drug-Resistant Infections. *Nano Today* **2012**, *7* (3), 201–222.

[28] Engler, A. C.; Tan, J. P. K.; Ong, Z. Y.; Coady, D. J.; Ng, V. W. L.; Yang, Y. Y.; Hedrick, J. L. Antimicrobial Polycarbonates: Investigating the Impact of Balancing Charge and Hydrophobicity Using a Same-Centered Polymer Approach. *Biomacromolecules* **2013**, *14* (12), 4331–4339. https://doi.org/10.1021/bm401248t.

[39] Qiao, Y.; Yang, C.; Coady, D. J.; Ong, Z. Y.; Hedrick, J. L.; Yang, Y.-Y. Highly Dynamic Biodegradable Micelles Capable of Lysing Gram-Positive and Gram-Negative Bacterial Membrane. *Biomaterials* **2012**, *33* (4), 1146–1153. https://doi.org/10.1016/j.biomaterials.2011.10.020.

[51] Leong, J.; Yang, C.; Tan, J.; Tan, B. Q.; Hor, S.; Hedrick, J. L.; Yang, Y. Y. Combination of Guanidinium and Quaternary Ammonium Polymers with Distinctive Antimicrobial Mechanisms Achieving a Synergistic Antimicrobial Effect. *Biomater. Sci.* **2020**, *8* (24), 6920–6929. https://doi.org/10.1039/D0BM00752H.

[65] Chin, W.; Zhong, G.; Pu, Q.; Yang, C.; Lou, W.; De Sessions, P. F.; Periaswamy, B.; Lee, A.; Liang, Z. C.; Ding, X.; Gao, S.; Chu, C. W.; Bianco, S.; Bao, C.; Tong, Y. W.; Fan, W.; Wu, M.; Hedrick, J. L.; Yang, Y. Y. A Macromolecular Approach to Eradicate Multidrug Resistant Bacterial Infections While Mitigating Drug Resistance Onset. *Nat. Commun.* **2018**, *9* (1), 917. https://doi.org/10.1038/s41467-018-03325-6.
